# Construction and Validation of a Universal Applicable Prognostic Signature for Gastric Cancer Based on Seven Immune-Related Gene Correlated With Tumor Associated Macrophages

**DOI:** 10.3389/fonc.2021.635324

**Published:** 2021-06-11

**Authors:** Junyu Huo, Liqun Wu, Yunjin Zang

**Affiliations:** Liver Disease Center, The Affiliated Hospital of Qingdao University, Qingdao, China

**Keywords:** gastric cancer, immune, macrophages, prognostic, signature

## Abstract

**Background:**

Tumor-associated macrophages (TAMs) play a critical role in the progression of malignant tumors, but the detailed mechanism of TAMs in gastric cancer (GC) is still not fully explored.

**Methods:**

We identified differentially expressed immune-related genes (DEIRGs) between GC samples with high and low macrophage infiltration in The Cancer Genome Atlas datasets. A risk score was constructed based on univariate Cox analysis and Lasso penalized Cox regression analysis in the TCGA cohort (n=341). The optimal cutoff determined by the 5-year time-dependent receiver operating characteristic (ROC) curve was considered to classify patients into groups with high and low risk. We conducted external validation of the prognostic signature in four independent cohorts (GSE84437, n=431; GSE62254, n=300; GSE15459, n=191; and GSE26901, n=109) from the Gene Expression Omnibus (GEO) database.

**Results:**

The signature consisting of 7 genes (FGF1, GRP, AVPR1A, APOD, PDGFRL, CXCR4, and CSF1R) showed good performance in predicting overall survival (OS) in the 5 independent cohorts. The risk score presented an obviously positive correlation with macrophage abundance (cor=0.7, p<0.001). A significant difference was found between the high- and low-risk groups regarding the overall survival of GC patients. The high-risk group exhibited a higher infiltration level of M2 macrophages estimated by the CIBERSORT algorithm. In the five independent cohorts, the risk score was highly positively correlated with the stromal cell score, suggesting that we can also evaluate the infiltration of stromal cells in the tumor microenvironment according to the risk score.

**Conclusion:**

Our study developed and validated a general applicable prognostic model for GC from the perspective of TAMs, which may help to improve the precise treatment strategy of GC.

## Introduction

Gastric cancer (GC), as one the most common malignant tumors, is the third leading cause of cancer death in the world (1). In recent years, with the progress and development of treatment methods, such as perioperative treatment and the application of targeted drugs, the survival time of patients with gastric cancer has been improved to some extent, but the overall prognosis is still unsatisfactory (2). In view of this, how to improve the overall survival of patients with gastric cancer is still a hot topic in the current research field.

The tumor microenvironment (TME) plays a critical role in tumorigenesis and development (3). Tumor-associated macrophages (TAMs), as an important component of the TME of malignant tumors (4), have been shown to have significant functions in the progression of malignant tumors, such as regulating the proliferation, invasion and metastasis of tumor cells (5–7). The degree of TAM infiltration is also directly related to the depth of tumor invasion, lymph node status and clinical stage of gastric cancer (8–10) and has become a new therapeutic target and prognostic indicator in the individualized treatment of gastric cancer. At present, the detailed mechanism of TAMs in gastric cancer is still not fully explored.

Effective prognosis evaluation is an important guarantee for the precise treatment of gastric cancer patients; however, the prognostic biomarkers that can be used in clinical practice are still limited to date. Considering the great potential of TAMs in prognosis assessment and precise targeting for gastric cancer treatment, the identification of specific markers of TAMs through high-throughput sequencing data may provide a valuable reference for new clinical diagnosis and treatment strategies of gastric cancer.

In this work, we explored the association between the infiltration abundance of macrophages and immune-related gene expression. Importantly, we constructed a prognostic model of gastric cancer based on seven immune genes related to macrophage infiltration and confirmed its prognostic value in different cohorts, which will help to formulate an individualized treatment plan for gastric cancer patients.

## Materials and Methods

### Data Acquisition

**Figure 1:**
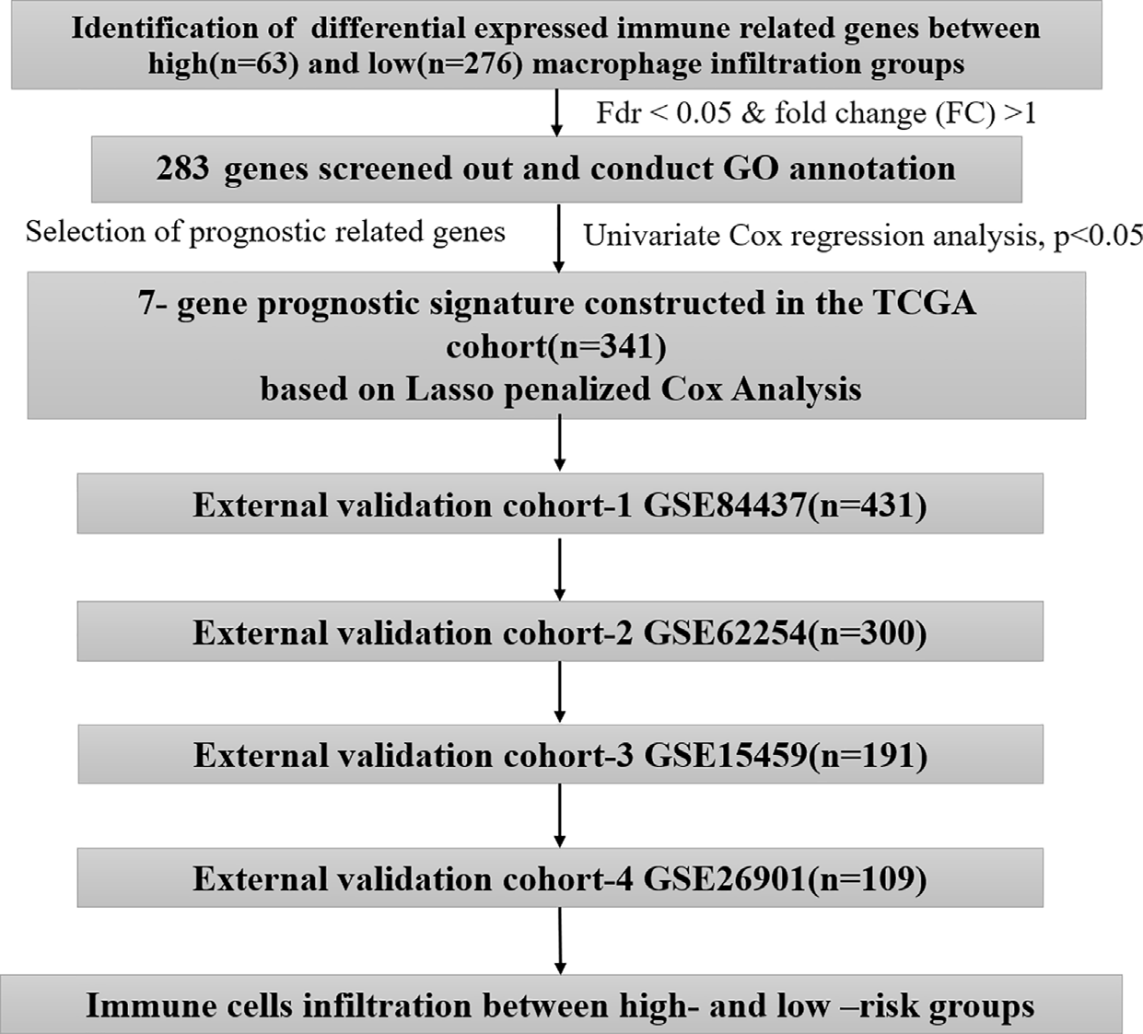
The workflow chart of this study.

We first obtained the immune infiltration data of The Cancer Genome Atlas (TCGA) from Tumor IMmune Estimation Resource Web Server (TIMER, https://cistrome.shinyapps.io/timer/) (11, 12). Then, we downloaded the gene expression profiles and corresponding clinical information from The Cancer Genome Atlas (TCGA, https://portal.gdc.cancer.gov/). A total of 341 GC patients with complete data were included in this study. The immune-related gene list was acquired from the ImmPort database (https://immport.niaid.nih.gov). Next, we acquired the gene expression profiles and the clinical data of four independent cohorts (GSE84437, n=431; GSE62254, n=300; GSE15459, n=191; and GSE26901, n=109) from the Gene Expression Omnibus (GEO) database (https://www.ncbi.nlm.nih.gov/geo/). The batch effects in different datasets were removed by the “ComBat” function of the R package “sva” (13). We complied with the access rules of the corresponding database during the process of data acquisition. Approval from the local ethics committee was not needed in this work because the above data were acquired from public databases. The workflow of this study and the clinical information of the above 5 independent cohorts are shown in **Figure 1** and **Table 1**, respectively.

**Table 1:** The clinical data of the 5 independent cohorts.

	TCGA (n=341)	GSE84437 (n=431)	GSE62254 (n=300)	GSE15459 (n=191)	GSE26901 (n=109)
Survival status					
alive	200	224	148	96	54
dead	141	207	152	95	55
Age					
>65	184	150	97	105	24
<=65	154	283	136	87	85
gender					
female	115	137	74	67	40
male	223	296	159	125	69
grade					
G1-2	131				
G3	201				
stage T					
T1-2	88	49			
T3	156	92			
T4	93	292			
stage N					
N0	100	80			
N1	91	188			
N2	71	132			
N3	68	33			
stage M					
M0	304				
M1	23				
stage TNM					
I-II	152		139	60	58
III	140		75	72	36
IV	35		19	60	15
Laurenclassification					
Diffuse		102	122	75	11
Intestinal		119	105	99	82
Mixed		10	6	18	5
Perineural Invasion					
YES			86		
NO			147		
lymphovascular					
YES			171		
NO			62		
Subtype					
Invasive				51	
Metabolic				40	
Proliferative				70	
Unstable				31	
stage M					
M0	304				102
M1	23				7
Adjuvant.chem					
YES					39
NO					70
Location					
antrum					56
body					36
entire					4
fundus					13

### Exploration of the Prognostic Significance of Macrophage Infiltration in Patients With Gastric Cancer

A total of 341 GC patients were assigned to the high and low macrophage infiltration groups given the optimal cutoff value determined by X-title software (14), where the overall survival of the two groups was compared by Kaplan–Meier survival analysis. Statistical significance was set as a p value of the log rank test less than 0.05.

### Identification of Differentially Expressed Immune-Related Genes (DEIRGs) Between the High and Low Macrophage Infiltration Groups

We extracted immune-related genes from the TCGA dataset and identified the differentially expressed immune-related genes (DEIRGs) between the high and low macrophage infiltration groups by the “limma” R package. A false discovery rate (FDR) of <.05 and log FC >1 were considered to be significant.

### Gene Ontology Function Annotation of DEIRGs

We carried out Gene Ontology (GO) functional annotation of the DEIRGs between the high and low macrophage infiltration groups by the R package “clusterProfile”, including cellular component (CC), molecular function (MF), and biological process (BP).

### Development and Validation of an Immune-Related Gene Prognostic Signature

Univariate Cox regression analysis and Kaplan–Meier survival analysis were initially combined for the preliminary screening of prognosis-related immune genes (PRIGs). P<0.05 was considered to be significant. Afterwards, the least absolute shrinkage and selection operator (LASSO) algorithm was applied to reduce the scope of PRIGs. While the LASSO penalized Cox analysis was implemented, we subsampled the dataset 1000 times and selected the PRIGs over 900 repeated times. A subselection of PRIGs was detected as a result of a penalty proportional to their size to shrink the regression coefficient (15, 16). Genes with zero regression coefficients were excluded. After that, regression coefficients were applied to establish a prognostic risk score, which was derived from LASSO Cox regression analysis of each PRIG multiplied by the expression level of each PRIG. The GC patients were classified into low-risk and high-risk groups considering the optimal cutoff corresponding to the maximum AUC value of the 5-year time-dependent receiver operating characteristic (ROC) curve (17). The LASSO regression analysis was performed with the “glmnet” R package. Time-dependent ROC curves and Kaplan–Meier survival curves were generated with the R packages “survivalROC” and “survminer”. To test the independent prognostic value of the risk score, univariate and multivariate Cox regression analyses were carried out. The four independent cohorts (GSE84437, n=431; GSE62254, n=300; GSE15459, n=191; and GSE26901, n=109) were used for the external validation of the prognostic model’s performance.

### Estimation of Immune Cell Infiltration

The abundances of six immune infiltrates (B cells, CD4+ T cells, CD8+ T cells, neutrophils, macrophages, and dendritic cells) in samples in TCGA datasets were estimated by the TIMER algorithm. The relative proportions of 22 infiltrated immune cell types were quantified by the CIBERSORT algorithm for each sample (18, 19). P < 0.05 was used as the standard to filter the sample.

### Exploration of the Tumor Microenvironment in Different Risk Groups

We calculated the StromalScore (which captures the presence of stroma in tumor tissue), ImmuneScore (which represents the infiltration of immune cells in tumor tissue), and ESTIMATEScore (which infers tumor purity) of the sample contained in the five independent cohorts based on the ESTIMATE (Estimation of STromal and Immune cells in MAlignant Tumor tissues using Expression data) algorithm using the R package “estimate”. We calculated the normalized enrichment score (NES) to quantify immune cell infiltration and immune function by single sample gene set enrichment analysis (ssGSEA) using the “GSVA” R package. Independent-samples t tests were used to compare the differences between the high- and low-risk groups, and p < 0.05 was suggested to indicate statistical significance.

## Results

### High Infiltration by Macrophages Is Associated With Unfavorable Overall Survival

**Figure 2:**
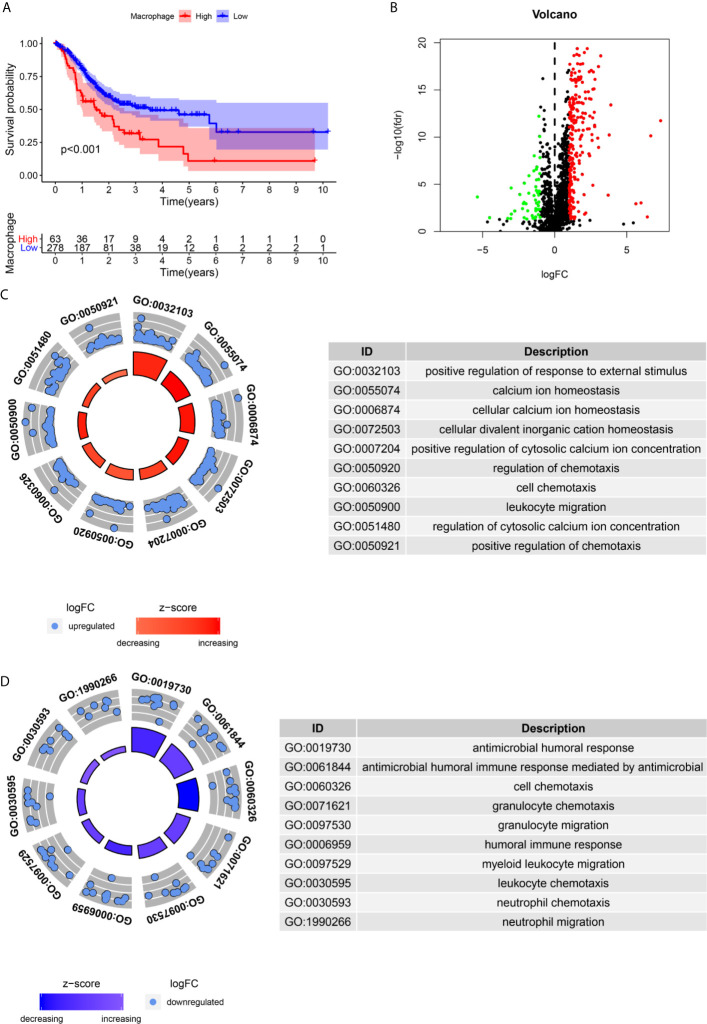
Identification of differential expressed immune-related genes(DEIRGs) associated with macrophage infiltration. **(A)** The high infiltration by Macrophages is unfavorable for the OS of GC. **(B)**. The vol plot DEIRGs. **(C)** The circle plot of GO terms up-regulated in the high macrophage infiltration group. **(D)** The circle plot of GO terms up-regulated in the low macrophage infiltration group.

We assessed the potential prognostic significance of immune cell infiltration for GC using the TIMER algorithm, and higher infiltration of macrophages was found to be associated with poor prognosis (**Figure 2A**). This provided an important basis for us to carry out subsequent research.

### Identification of DEIRGs Associated With Macrophage Infiltration

A total of 283 DEIRGs were identified by the Wilcox test in the “limma” R package (**Figure 2B**). A total of 234 genes were upregulated in the high macrophage infiltration group (log FC>1), and 49 genes were upregulated in the low macrophage infiltration group (log FC<-1) (**Figure 2B**).

### GO Enrichment Analysis for the DEIRGs

The immune-related genes upregulated in the high macrophage infiltration group were mainly enriched in calcium ion homeostasis, cellular divalent inorganic cation homeostasis, receptor ligand activity, leukocyte migration, etc. (**Figure 2C**). The immune-related genes upregulated in the low macrophage infiltration group were mainly enriched in receptor ligand activity, cell chemotaxis, cytokine receptor binding, and antimicrobial humoral response (**Figure 2D**).

### Establishment of a Seven-Immune Gene Prognostic Signature in the TCGA Cohort

**Figure 3:**
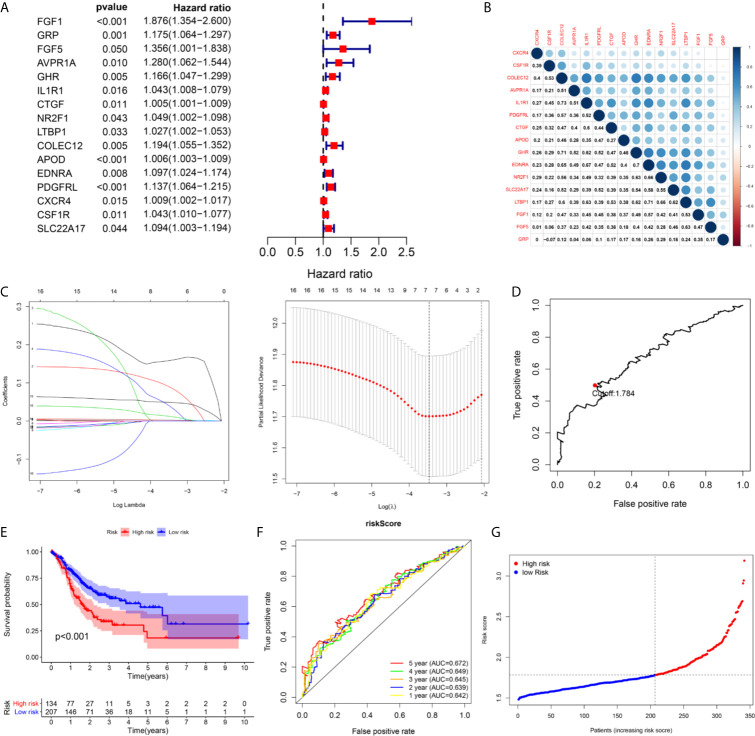
The building process of the seven immune gene prognostic signature in the TCGA cohort. **(A)** The forrest plot of the univariate Cox analysis. **(B)** The corrplot of the prognostic related genes. **(C)** Lasso penalized COX regression analysis. **(D)** The optimal cutoff determined by 5-year time-depend ROC curve. **(E, F)** The Kaplan–Meier survival analysis and time‑dependent ROC analysis of the signature for predicting the OS of patients in the TCGA cohort. **(G)** The risk score distribution of patients in in the TCGA cohort.

**Figure 4:**
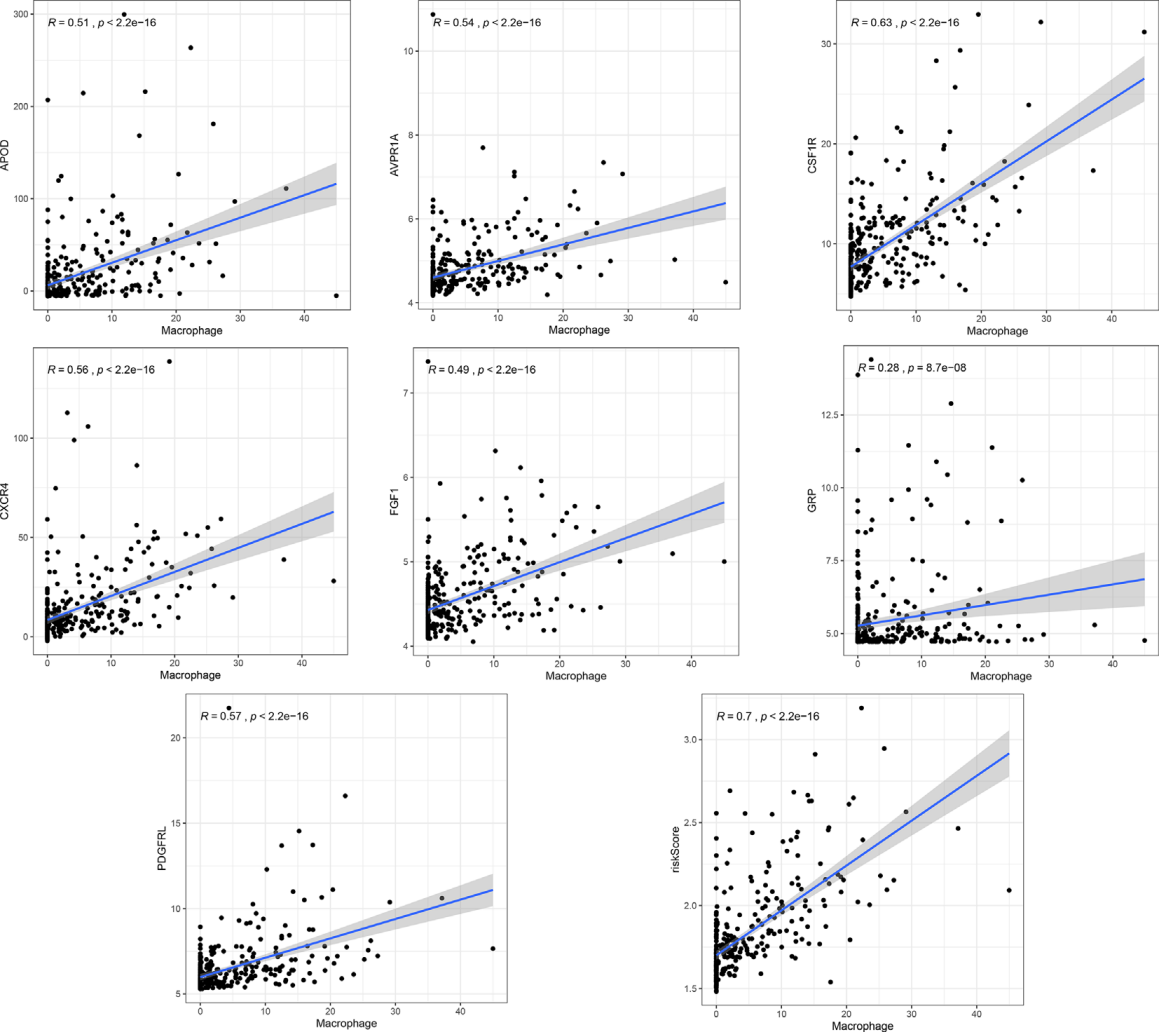
The correlation analysis of the signature and the macrophage infiltration.

**Figure 5:**
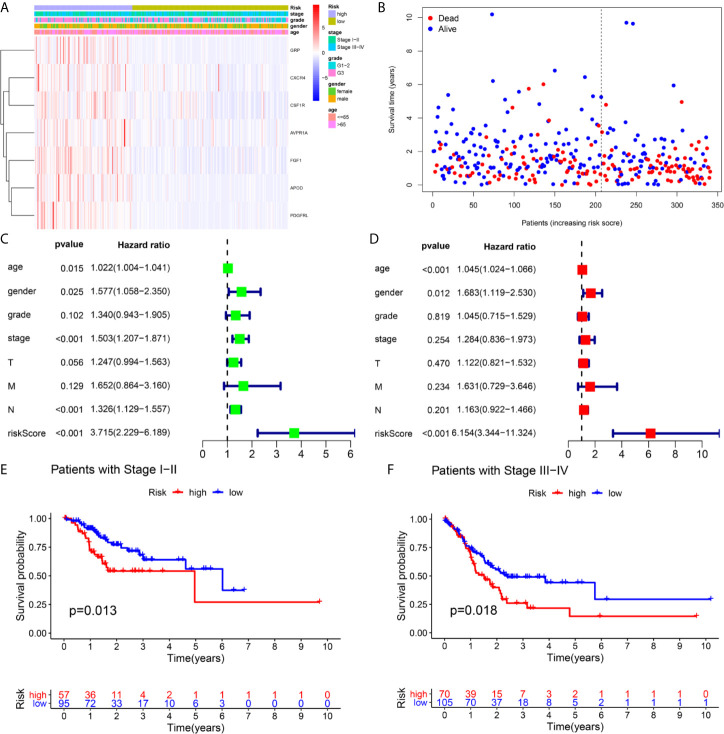
Independence validation of the risk score in the TCGA cohort. **(A, B)** The heatmap, and the survival status of patients in in the TCGA cohort. **(C)** The forrest plot of the univariate Cox analysis. **(D)** The forrest plot of the multivariate Cox analysis. **(E, F)** Subgroup validation based on the clinical stage.

A total of 16 genes were selected as prognosis-related genes by univariate Cox regression analysis and Kaplan–Meier survival analysis (**Figures 3A, B**). LASSO Cox regression analysis was then applied to exclude genes that may be highly correlated with other genes (**Figure 3C**). We ultimately identified a 7-gene signature. The risk score = FGF1*0.1606 + GRP*0.0835 + AVPR1A*0.0316 + APOD*0.0024 + PDGFRL*0.0482 + CXCR4*0.0019 + CSF1R*0.0108. Patients were assigned into low-risk and high-risk groups according to the optimal cutoff of 1.784 determined by the 5-year ROC curve (**Figures 3D, G**). The results showed that the overall survival rate (OS) of the high-risk group was significantly lower than that of the low-risk group (**Figure 3E**). The area under the curve (AUC) values for the model predicting OS at 1, 3 and 5 years were 0.642, 0.645 and 0.672, respectively (**Figure 3F**). It is worth mentioning that the expression levels of 7 genes in the signature were positively correlated with macrophage abundance, as verified by Spearman correlation analysis (**Figure 4**), indicating that we could estimate the degree of macrophage infiltration in GC tissue according to the risk score. The risk of death of GC patients increased with the increasing risk score (**Figures 5A, B**). Then, we included the risk score and other clinical factors in univariate and multivariate Cox regression analyses, and the results showed that the risk score was an independent prognostic indicator (**Figures 5C, D**). The prognostic signature was applicable for GC patients in early and advanced stages (**Figures 5E, F**).

### External Validation of the Prognostic Signature in Four Independent Cohorts

**Figure 6:**
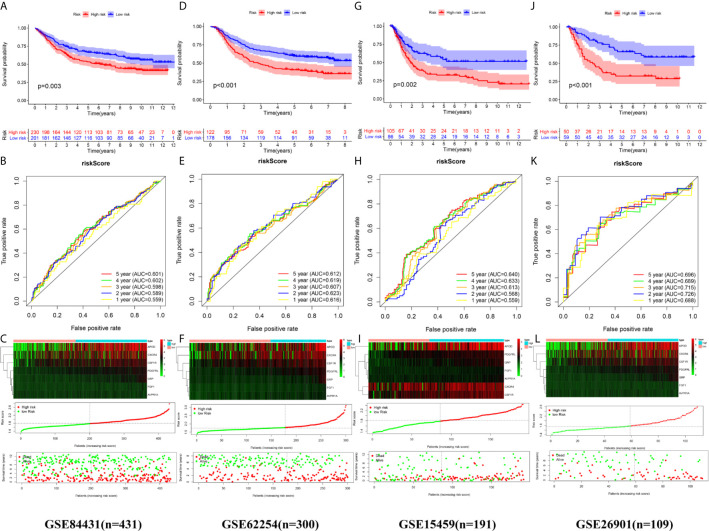
External validation of the prognostic model. **(A, B)** The Kaplan–Meier survival analysis and the time‑dependent ROC analysis of the signature for predicting the OS of patients in the GSE84437 cohort. **(C)** The heatmap, distribution of risk score, and the survival status of patients in in the GSE84437 cohort. **(D, E)** The Kaplan–Meier survival analysis and the time‑dependent ROC analysis of the signature for predicting the OS of patients in the GSE62254 cohort. **(F)** The heatmap, distribution of risk score, and the survival status of patients in in the GSE62254 cohort. **(G, H)** The Kaplan–Meier survival analysis and the time‑dependent ROC analysis of the signature for predicting the OS of patients in the GSE15459 cohort. **(C)** The heatmap, distribution of risk score, and the survival status of patients in in the GSE15459 cohort. **(J, K)** The Kaplan–Meier survival analysis and the time‑dependent ROC analysis of the signature for predicting the OS of patients in the GSE26901 cohort. **(L)** The heatmap, distribution of risk score, and the survival status of patients in in the GSE26901 cohort.

**Figure 7:**
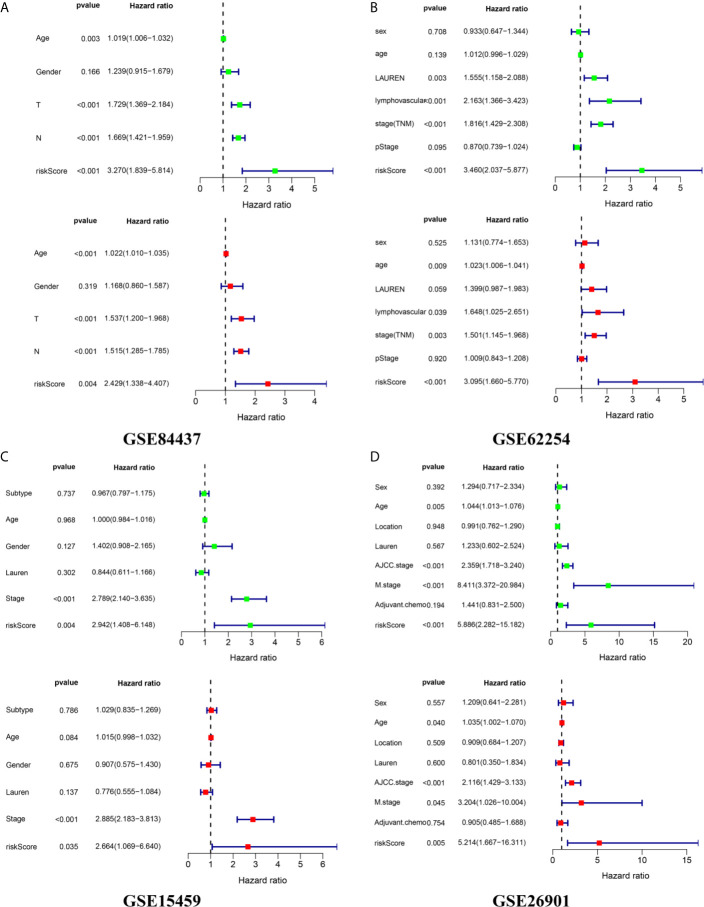
External independence validation of the prognostic model in the **(A)** GSE84437 cohort **(B)** GSE62254 cohort **(C)** GSE15459 cohort **(D)** GSE26901 cohort. *green represents the univariate Cox analysis, red represents the multivariate Cox analysis.

We calculated the risk score of each sample in the four independent cohorts using the calculation formula derived from TCGA and assigned them into groups with a high risk or low risk based on the unified cutoff consistent with the TCGA cohort. The results of survival analysis revealed that the OS of patients in the high-risk group was significantly shorter than that in the low-risk group in each independent cohort (**Figures 6A, D, G, J**). In the GSE84437 cohort, the AUC values for the risk score predicting OS at 1, 3 and 5 years were 0.559, 0.598 and 0.601, respectively (**Figure 6B**). In the GSE62254 cohort, the AUC values for the risk score predicting OS at 1, 3 and 5 years were 0.616, 0.607 and 0.612, respectively (**Figure 6E**). In the GSE15459 cohort, the AUC values for the risk score predicting OS at 1, 3 and 5 years were 0.559, 0.613 and 0.640, respectively (**Figure 6H**). In the GSE26901 cohort, the AUC values for the risk score predicting OS at 1, 3 and 5 years were 0.688, 0.715 and 0.696, respectively (**Figure 6K**). The patients’s risk death was positively correlated with the risk score (**Figures 6C, F, I, L**). The results of univariate and multivariate Cox regression analysis confirmed that the risk score was an independent prognostic indicator in each independent cohort (**Figures 7A–D**). These results demonstrated the robustness of this prognostic model.

### Immune Cell Infiltration Between Different Risk Groups

**Figure 8:**
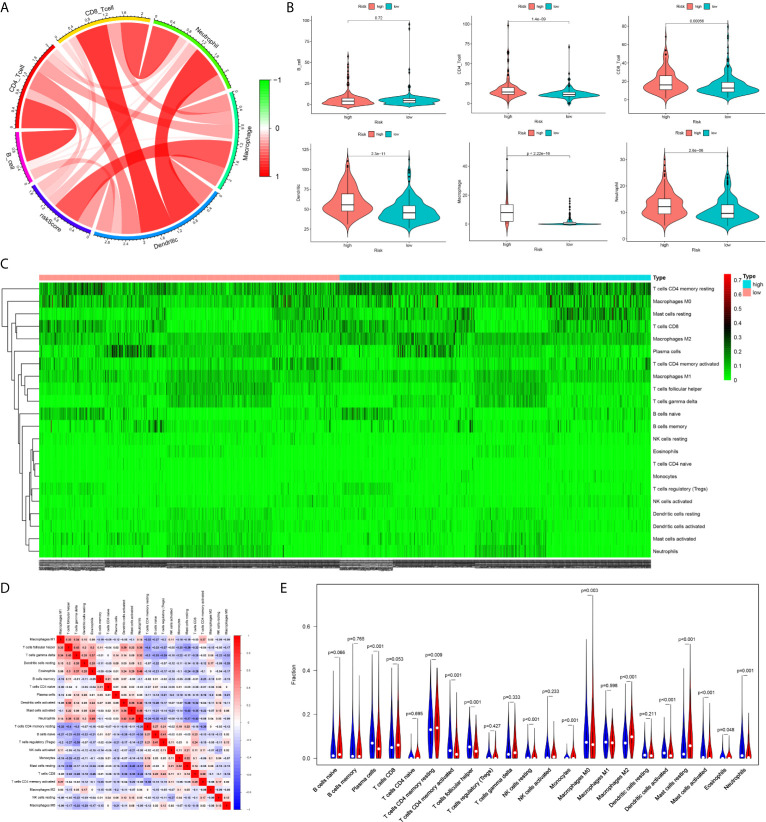
The difference of immune cell infiltration between high- and low-risk groups. **(A)** The circos plot of risk score and the infiltration of six types of immune cells. **(B)** The vioplot showed the difference of the abundances of six immune infiltrates (TIMER algorithm) between high- and low risk groups in the TCGA cohort. **(C)** The heatmap of 22 kinds of immune cells infiltration. **(D)** The corHeatmap of 22 kinds of immune cells infiltration. **(E)** The vioplot showed the difference of the abundances of 22 types of immune cells infiltrates between high- and low risk groups[CIBERSORT algorithm, red represent high risk(n=527), blue represent low risk(n=509)].

The infiltration levels of CD4+ T cells, CD8+ T cells, neutrophils, macrophages, and dendritic cells in the high-risk group were all higher than those in the low-risk group estimated by the TIMER algorithm (**Figures 8A, B**). The infiltration levels of resting memory CD4+ T cells, resting mast cells, and M2 macrophages in the high-risk group were higher than those in the low-risk group estimated by the CIBERSORT algorithm. The infiltration levels of plasma cells, activated memory CD4+ T cells, follicular helper T cells, and M0 macrophages in the low-risk group were higher than those in the high-risk group estimated by the CIBERSORT algorithm (**Figures 8C–E**).

### The Relationship Between the Tumor Microenvironment and the Prognostic Signature

**Figure 9:**
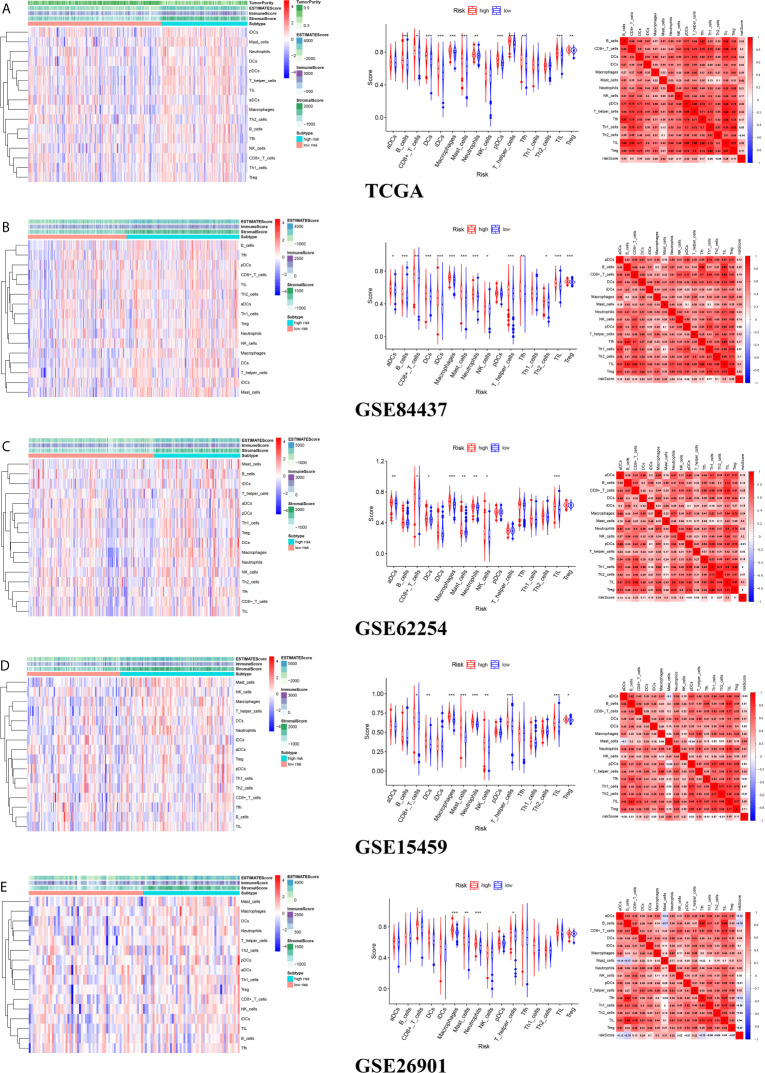
The landscape of tumor microenvironment for difference of immune cell infiltration between high- and low-risk groups **(A)** TCGA cohort **(B)** GSE84437 cohort **(C)** GSE62254 cohort **(D)** GSE15459 cohort **(E)** GSE26901 cohort.

**Figure 10:**
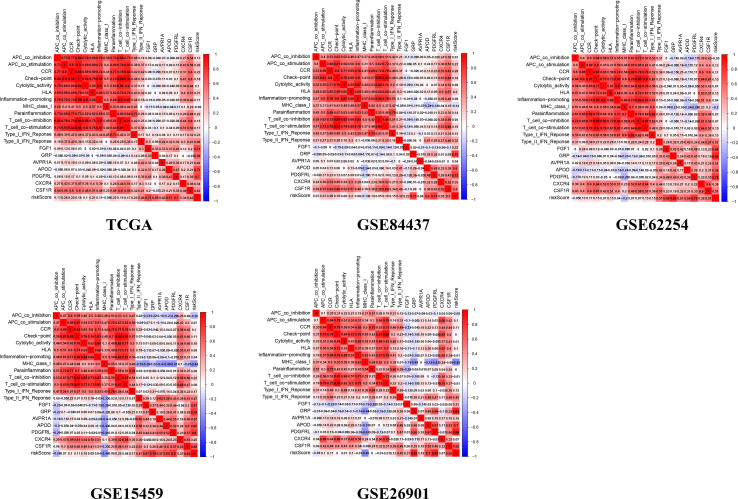
The correlation between genes and immune function.

**Figure 11:**
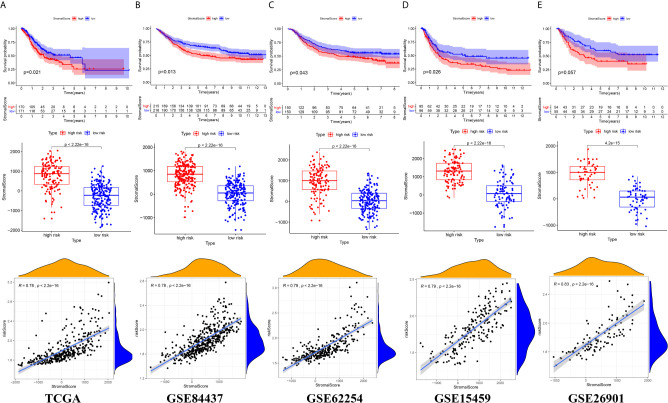
The correlation between risk score and Stromal score **(A)** TCGA cohort **(B)** GSE84437 cohort **(C)** GSE62254 cohort **(D)** GSE15459 cohort **(E)** GSE26901 cohort.

There were common differences between the high- and low-risk groups in the 5 independent cohorts. For example, the abundances of macrophages, mast cells, and neutrophils in the high-risk group were all higher than those in the low-risk group (**Figures 9A–E**). The upregulation of CXCR4 and CSF1R expression is related to the enhancement of multiple immune functions, such as T cell costimulation and coinhibition, checkpoints, and CCR, while the upregulation of FGF1, GRP, AVPR1A, APOD, and PDGFRL was associated with the weakening of T cell APC coinhibition and MHC class I (**Figure 10**). Another important finding was that a higher StromalScore was found to be associated with an unfavorable prognosis of GC (**Figures 11A–E**). Interestingly, the risk score was highly positively correlated with the StromalScore in the five independent cohorts (**Figures 11A–E**), which may help us to explain the causes leading to different clinical outcomes in different risk groups.

## Discussion

Gastric cancer (GC) is a digestive tract malignant tumor with a high incidence rate and mortality (20). Surgery combined with radiotherapy and chemotherapy is the main method for the treatment of GC. However, because of the occult early symptoms of GC, most patients are initially diagnosed in the middle and advanced stages, with a 5-year survival rate of less than 20% (21). The traditional TNM staging system seems to have difficulty accurately assessing the overall prognosis of GC patients (22). For example, in clinical work, we will find that some GC patients with early pathological stages may not have a high overall survival rate. In recent years, with the development of research on the pathogenesis of GC, an increasing number of surgeons have realized that the factors that determine the survival time of GC patients after surgery are not only the complete resection of the tumor but also the gene expression of the tumor (23, 24).

As an important component of the tumor microenvironment (TME), tumor-associated macrophages (TAMs) play a critical role in the tumorigenesis and development of GC (25–27) and have become a new therapeutic target and prognostic indicator in GC. Recent studies have shown that TAMs can promote tumor progression by participating in the immune regulation of GC (28–30). The exploration of immune genes associated with TAMs may provide new biomarkers for the prognostic assessment of GC.

Considering that research on TAMs and immune gene expression is still lacking in the field of GC, we conducted this study. We confirmed the prognostic value of TAMs for GC, and GC patients with higher macrophage infiltration were found to have a poor prognosis. Then, we found that there were significant differences in the expression of immune-related genes between the high and low macrophage infiltration groups, and the functions of the immune genes that were upregulated in the two groups were also different, indicating that macrophage abundance did have a certain impact on the immunophenotype of GC. Whether this is the direct cause of the difference in prognosis between the two groups is still unknown. However, such a finding provides an important hypothesis; that is, TAMs may lead to different clinical outcomes of GC indirectly by influencing the immunophenotype of GC. Next, we screened seven genes from these differentially expressed genes to form the risk score. Interestingly, an obviously positive correlation was observed between the risk score and macrophage abundance, implying that we can speculate what the level of macrophage infiltration is in GC based on the risk score. At present, TAMs have been considered a new target for the treatment of GC (31), and this discovery will undoubtedly provide important clues for treatment strategies focusing on TAMs. In addition, the abundance of macrophages in GC tissues was estimated by targeted sequencing of seven specific genes, which will also maximize cost-effectiveness.

We conducted external validation in four independent cohorts to test the reliability of the prognostic model. The results showed that we could accurately identify GC patients with good and poor prognoses depending on the model, which means that clinicians can provide individualized treatment for GC patients based on the risk score. For example, for patients with high risk scores, clinicians should closely follow up and make corresponding postoperative review plans, while for patients with low risk scores, excessive treatment should be avoided, which is of great significance for reasonable allocation of medical resources. Current studies have shown that the TAM cell population is in a state of continuous transformation between M1 and M2 macrophages, and M2 macrophages have immunosuppressive and tumor-promoting effects (32). We found that the macrophage M2 infiltration level in the high-risk group was significantly higher than that in the low-risk group, which may be an important factor for the poor prognosis of the high-risk group. Meanwhile, it also suggested that the risk score may play a critical role in the process of TAM phenotype polarization to the M2 type. Apart from macrophages, the high-risk group also exhibited a higher abundance of mast cells and neutrophils. According to related reports, mast cells in the TME can release VEGF to support tumor angiogenesis and degrade extracellular matrix by releasing matrix metalloproteinase-9 (MMP9) to promote metastasis, which is conducive to tumor progression (33). Transforming growth factor-β (TGF-β) in the TME could promote neutrophil polarization to the N2 type, which could stimulate immunosuppression, tumor angiogenesis, proliferation, and metastasis (34–36).

Stromal cells in the TME can be divided into angiogenic vascular cells (AVCs), cancer-associated fibroblasts (CAFs), cancer-associated adipocytes (CAAs), and mesenchymal stromal cells (MSCs) (37). AVCs provide nutrition and oxygen for tumor cells, remove metabolic waste, and provide an entry point for metastatic tumor cells to enter the circulatory system (38). In the TME, CAFs promote the formation of an oxygen-rich, immunosuppressive and proinflammatory microenvironment and indirectly support tumor occurrence (39). CAAs provide energy for the growth of tumor cells by producing metabolites and lipid factors that promote tumor growth, promote the invasion characteristics of tumor cells in the primary tumor site and distant metastasis, and protect tumor cells from the influence of various treatments (40, 41). MSCs promote tumor angiogenesis by secreting angiogenic factors and promoting perivascular tissue differentiation into pericytes and promote tumor cell movement and metastasis to distant organs by producing CCL5 (42). Therefore, targeted therapy of stromal cells in the TME will have a positive impact on the prognosis of cancer patients. Considering the close correlation between the risk score and stromal cells, the seven genes in the signature may be a new target for the treatment of stromal cells.

Overexpression of fibroblast growth factor 1 (FGF1) is observed in various cancers and is correlated with poor survival (43). Depletion of AVPR1A in castration-resistant prostate cancer cells resulted in decreased cell proliferation and reduced cyclin A (44). Apolipoprotein D (APOD) has been determined to be a predictor of breast cancer recurrence among tamoxifen-treated patients with estrogen receptor positivity (ER+) (45). The modulation of platelet-derived growth factors (PDGFs) and their receptors (PDGFRs) through overexpression and silencing is widely used in cancers and is attractive as an oncologic target with diverse therapeutic possibilities, leading to a role as a clinical variable and in the nodal metastasis of GC (46). CXC type 4 chemokine receptor (CXCR4), synonymous with fusion protein (Fusin) or CD184, plays a role in promoting migration and mediating cell death regulated by autophagy in the peritoneal diffusion of gastric cancer cells (47). Zhu (48) demonstrated *in vivo* that interrupting signaling by the myeloid growth factor receptor CSF1R in a mouse model of pancreatic ductal adenocarcinoma (PDAC) can effectively reprogram macrophage reactions, causing enhanced antigen presentation and antitumor T cell responses.

Our study is the first to clarify the prognosis and application value of immune-related genes in gastric cancer from the perspective of TAMs. The establishment and validation of the prognostic model were based on 5 independent cohorts, with a total of 1372 patients, which is the largest prognostic model discovery project for GC so far. Our work has produced some convincing results, but there are still some deficiencies that need to be improved or supplemented in the future. For example, the specific mechanism of the seven genes contained in the signature in GC is still unclear and needs further exploration.

## Conclusion

Our study developed and validated a general applicable prognostic model for GC from the perspective of TAMs, which may help to improve the precise treatment strategy of GC.

## Data Availability Statement

Publicly available datasets were analyzed in this study. This data can be found here: The datasets analyzed for this study were obtained from The Cancer Genome Atlas (TCGA, https://portal.gdc.cancer.gov/), Tumor IMmune Estimation Resource Web Server (TIMER, https://cistrome.shinyapps.io/timer/), and Gene Expression Omnibus (GEO, https://www.ncbi.nlm.nih.gov/geo/).

## Author Contributions

JH and LW designed this study. JH analyzed the data in this study and interpreted the findings and drafted the manuscript. JH, LW, and YZ carried out data management and revised the manuscript. All authors contributed to the article and approved the submitted version.

## Conflict of Interest

The authors declare that the research was conducted in the absence of any commercial or financial relationships that could be construed as a potential conflict of interest.

## Abbreviations

GC, gastric cancer; TCGA, The Cancer Genome Atlas; TIMER, Tumor IMmune Estimation Resource; GEO, Gene Expression Ominibus; TME; Tumor microenvironment; TAMs, Tumor associated macrophages; DEIRGs: differential expressed immune-related genes; GO, Gene ontology; CC, cellular component; MF, Molecular Function; BP, biological process; LASSO, least absolute shrinkage and selection operator; PRIGs: prognostic related immune genes; ROC, receiver operating characteristic; AUC, area under curve; OS, overall survival. ESTIMATE, Estimation of STromal and Immune cells in MAlignant Tumor tissues using Expression data; NES, normalized enrichment score; ssGSEA, single sample gene set enrichment analysis; TME, tumor microenvironment; AVC, giogenic vascular cell; CAF, cancer associated fibroblast; CAA, cancer-associated adipocyte cell; MSC, mesenchymal stromal cell.
